# Hospital Meetings, &c.

**Published:** 1898-12-24

**Authors:** 


### HOSPITAL MEETINGS, &C.

### THE PRINCE OF WALES'S HOSPITAL FUND FOR LONDON.

A meeting of the General Council of the Prince of Wales's Hospital Fund for London was held at Marlborough House on Tuesday, 20th inst., H.R.H. the Prince of Wales in the chair. Amongst others there were present:—The Chief Rabbi, Lord Rowton, Lord Farquhar, Mr. Hugh Colin Smith, Sir Samuel Wilks, Bart. (President of the Royal College of Physicians), Right Hon. C. Stuart Wortley, Q.C., M.P., Sir Trevor Lawrence, Bart., Sir Henry Burdett, K.C.B., Mr. John Aird, M.P., Mr. E. A. Hambro, Mr. Albert C. Sandeman, Mr. Julius Wernher, Sir Savile Crossley, Bart. and Mr. J. G. Craggs (hon. secretaries). Letters regretting their inability to attend were received from the following: Duke of Westminster, Duke of Norfolk, Lord Rothschild (treasurer), Lord Iveagh, Lord Reay, Mr. Sydney Buxton, M.P., and the Rev. T. Bowman Stephenson.

Report of the Executive Committee.

The report of the Executive Committee, which contained a full financial statement of the present condition of the Fund, was presented. The receipts for the current year up to December 17th were shown to have been £38,494, which, with the balance of the funds for 1897, gave a total sum in hand of £203,490. Having regard to all the circumstances, the Executive Committee were of opinion that £32,500 was available for distribution this year.

Report of the Distribution Committee.

The report of the Distribution Committee, which was to the following effect, was appended:—

The Committee, in presenting their report, think it desirable to refer briefly to the altered conditions attending the distribution of the Fund this year as compared with last.

Last year time did not admit of the personal visitation of any hospitals, and even inquiry by letter into the circumstances and special needs of the several institutions had to be limited to those which had 100 or more beds occupied daily on an average taken throughout three years. To most of these chief hospitals annual grants were made out of the income of the Fund, conditional on the amount being used in each case for supplying some special needs, such as the opening of closed wards, the improvement of the accommodation for the nursing staff, the remedying of structural defects, or the relief of serious financial embarrassment. The principal hospitals which received no annual grants were the few whose resources were such that they did not appear to stand in need of special assistance.

Last year there was at the disposal of the Fund a considerable sum which had resulted from the sale of the Hospital stamps, and it was decided, with the sanction of his Royal Highness, to devote it to special Jubilee donations, following the practice of the Hospital Sunday Fund both as regards the institutions which should be aided, and the amount given in each case. This year, in the absence of any substantial sum derived from Stamps, the amount available for distribution is much less, being only what may be fairly regarded as the present income of the Fund, in the shape of annual contributions and interest on invested capital. The system of distribution, however, has undergone a great improvement.

A large Visiting Committee was appointed in the summer with the approval of his Royal Highness, consisting of physicians and surgeons, of wide experience, and about an equal number of laymen specially interested in hospital management. Sir Trevor Lawrence, having been elected chairman, the Committee was divided into sub-committees of two or three each, having in every case at least one medical member, and such of the metropolitan hospitals concerned as lay within a radius of seven miles of Charing Cross were distributed systematically among the sub-committees, and all were submitted to thorough inspection and inquiry. The reports of the various sub-committees being then tabulated under appropriate headings, most valuable and reliable information has been placed at the disposal of the Distribution Committee.

Before proposing renewal of the annual grants to the principal hospitals, the Committee made inquiry as to how far the conditions on which they were given had been complied with; and the answers having proved very satisfactory we recommend that these annual grants be continued this year, and in the same amounts as before; except that in the present state of the Fund we do not advise that more than £5,000 be given annually to any hospital.

These annual grants to the principal hospitals absorb £20,450, which deducted from £32,460, the estimated present income of the Fund, leaves £12,000 available for distribution otherwise. We recommend that this sum be employed for distribution, as a rule, for donations, as distinguished from annual grants. But to this rule we recommend exceptions in certain cases, with the view of opening wards now closed. The result will be that 37 beds now unoccupied will come into use during the next year, and if we add the 185 beds reoccupied last year we have a total of 242 beds made permanently available to the public without payment as one result of the operation of the Fund hitherto. This must in itself be regarded as an immense boon to the suffering poor of the metropolis.

The Committee recommend that the 100 dozen pints of champagne given by Messrs. Charles Heidsieck, be distributed among the hospitals, receiving grants approximately in proportion to the number of beds occupied, exclusive of the pay beds.

Report of the Convalescent Hospitals Committee.

The report of the Committee appointed to distribute the sum given to convalescent hospitals was also attached, and was as follows:—

The application of the sum of £1,000 contributed by the Trustees of the London Parochial Charities (City Parochial Foundation), for the benefit of convalescent hospitals, was referred to a special committee, having particular relations with the City, together with the honorary secretaries and Dr. Freshfield, one of the trustees. The Committee were bound to consider the qualifications of applicants in respect first of their connexion with London, and secondly of their coming fairly within the definition of convalescent hospitals. The sum to be distributed not being very large the Committee have thought it better to allocate it among a limited number in fairly substantial grants rather than to fritter it away in small sums.

The report of the Executive Committee having been adopted,

H.R.H. the Prince of Wales said: I have now to return my warmest thanks not only to those gentlemen who have served on the Visiting Committee but also to the members of the Distribution and Convalescent Hospitals Committee. I am well aware that they have given much time and trouble to the consideration of the matters set forth in the report, and that they have gone carefully into the question of the grants to be made to the different hospitals. So far as I can judge, the money at their disposal has been well distributed.

Sir Henry Burdett said that people had expressed a doubt as to the effect the Prince of Wales's Fund had had on the hospitals of the metropolis. So far from this effect having been harmful, it had led something like 1,100,000 people to give to the fund in the first year of its existence, and of these some 400,000 seemed to be likely to become regular contributors. Besides this, the income of the 93 metropolitan hospitals in 1897 showed an increase, compared with the average receipts of the three previous years, of no less than £150,000. The ordinary income increased by £35,000, legacies by £62,000, and donations for special purposes by upwards of £55,000. Nor was this all, for whereas the legacies in 1895 were about the same as those received in 1897, the special donations given to the hospitals in 1897 exceeded those given in 1894 by £65,000, those given in 1895 by £32,000, and those given in 1896 by £69,000.

A vote of thanks to H.R.H. the Prince of Wales for presiding was unanimously passed on the motion of Lord Farquhar, and the meeting terminated.

The grants will be transmitted to the various institutions on December 30th, and a detailed account of their allocation will be communicated to the press on the same day.

### VICTORIA HOSPITAL FOR CHILDREN.

Her Royal Highness Princess Louise was present at a well-attended meeting held on Monday, 12th inst., at Grosvenor House, by permission of the Duke of Westminster, on behalf of the new building scheme of the Victoria Hospital for Children. The proposed building, to be erected near Kennington Oval at an estimated cost of £25,000, will supply the whole accommodation for in-patients, and will provide six wards on three floors, each containing 16 beds—96 in all—with plenty of ventilation and cubic air space. Each ward is to be self-contained, and able to be closed for disinfection without interfering even with the other ward on the same floor. There will also be accommodation for anæsthetic and operating rooms, and for the isolation of infectious cases, with no direct communication with the rest of the building, and approached by a separate staircase; and in the basement improved domestic arrangements, including a good dining-room for nurses, store-rooms, and so forth; while the existing building will be adapted to provide much needed accommodation for management, staff, and other purposes.

The Duke of Westminster, who took the chair in the absence of Earl Cadogan, the president of the institution, said he hoped that inaugural meeting would result in adequate support being given to the committee in the step they were taking after the most careful deliberation.

Mr. Martin Ridley Smith, the chairman of the general committee of the hospital, explained in detail the reasons which had induced the committee to decide to incur the large expense involved in the new scheme. The existing building was never intended for a hospital; it was an ordinary dwelling-house, quite unsuited to its present use. Undoubtedly, much excellent work had been done within its walls, but in the light of the knowledge acquired of late years about hospitals, and in the region of sanitary science, it was safe to say that that work would have been more satisfactory and successful had they not been so terribly hampered by their surroundings. The four principal wards were set in pairs on two floors; they lay close alongside each other, and were in direct communication. This was manifestly a most unfortunate arrangement, because if any infectious case occurred in one ward both of them—constituting half their effective space—had to be closed. No alteration in this respect was possible, as, owing to the structural necessities of the building one ward must always serve as a passage to another. Then the wards were insufficiently and unscientifically ventilated. There should be what was known as cross ventilation, but theirs was only from end to end, with the result that the air round the cots was too stagnant. Cases had occurred in which the physician had ordered a child's bed to be placed in the centre of the room, so that the patient might get more air. Another defect—a serious and dangerous one—was that the double wards had but one kitchen, bath-room, and lavatory between them, and these in immediate communication with the wards. The sanitary condition of these offices was as good as it could be made, but they ought to be separated from the wards by at least a short cross-ventilated lobby; such an improvement was, however, absolutely impossible. The next point was that the cubic air space was insufficient, the ceilings being low. To remedy this they had reluctantly been obliged to reduce the number of beds by ten, and that just at a time when circumstances made them most anxious to largely increase their available accommodation. Most serious of all, however, was their lack of means to isolate infectious cases. True, they had a ward situated outside the hospital where two or three patients could be accommodated, but that had been built many years ago in the infancy of scientific knowledge upon the subject, and it was the deliberate opinion of the Medical and General Committees that it was quite unfit for the reception of patients. The Princess Louise, the patron of the hospital, had expressed her wish that when the plans of the new building were considered, the fullest possible provision should be made in this matter of isolation, and he could assure her Royal Highness that these views were absolutely in accordance with those of the committee, and that her wishes should receive the utmost care and attention. Finally, they had not sufficient accommodation for the staff. One of the resident medical officers had to sleep off the premises—a most undesirable arrangement—the secretary had no room to himself, one room having to serve as board-room, clerks' office, and dining-room, while the store-rooms were most insufficient. The fact of the matter was that the whole building was terribly overcrowded—full to overflowing from garret to basement. Could they wonder, then, that they were troubled with mysterious outbreaks of infectious diseases? The building was altogether too old. They must have a new building.

Mr. Pickering Pick, the senior medical officer, gave a very graphic and picturesque account of the state of things among the little patients, and emphasised the fact that the demands upon the hospital were continually increasing.

Mr. Alfred Farquhar, the treasurer, dealing with the financial position of the institution, said they had no endowments, and they were naturally most reluctant to realize their invested resources; they made their appeal with an empty exchequer and an attenuated balance-sheet. Up till that day the building fund had amounted to £1,100, but that sum had been increased to £2,000 by donations from members of the committee.

A vote of thanks having been passed to the Duke of Westminster for the use of Grosvenor House, his Grace, in reply, observed that the growth of the population of London—some 40,000 yearly—must give rise to anxiety with regard to hospital accommodation. Whether voluntary effort would be capable of coping with that increase was a matter about which they could not then decide.

